# Mesenteric Cyst: A Case Report of a Rare Entity

**DOI:** 10.7759/cureus.94731

**Published:** 2025-10-16

**Authors:** Diogo L Monteiro, Filipa Eiró, Helena Contente, Claudia Branco, Claudia Santos

**Affiliations:** 1 General Surgery, Unidade Local de Saúde de Lisboa Ocidental, Lisbon, PRT

**Keywords:** abdominal tumor, laparoscopy, mesenteric cyst, minimally invasive surgery, rare tumor

## Abstract

Mesenteric cysts are rare entities of the abdominal cavity, typically benign, that originate within the mesenteric tissue of the gastrointestinal tract. Their occurrence in adults is extremely uncommon and rarely encountered in clinical practice. These lesions may occur at any site along the mesentery, most often in the small bowel mesentery, and their clinical presentation varies from incidental findings to symptomatic abdominal pain, distension, or acute complications. Preoperative diagnosis can be challenging due to nonspecific symptoms, but advances in imaging modalities such as CT and MRI have facilitated more accurate identification, characterization, and surgical planning.

We report the case of a 63-year-old male who presented with isolated left-sided abdominal pain. Cross-sectional imaging demonstrated a 56 mm cystic lesion within the mesentery, located adjacent to the celiac trunk, a previously unreported site. The patient underwent elective laparoscopic excision of the cyst. The procedure was completed successfully without intraoperative or postoperative complications. Histopathological examination confirmed the diagnosis of a benign mesothelial inclusion cyst.

Mesenteric cysts require surgical excision as the treatment of choice, both to establish a definitive diagnosis and to prevent potential complications such as infection, hemorrhage, or obstruction. Minimally invasive approaches, including laparoscopy, have been shown to be safe and effective, offering shorter recovery times and reduced morbidity compared to open procedures. This case illustrates the clinical features, diagnostic pathway, and surgical management of a rare mesenteric cyst located near the celiac trunk, emphasizing the role of imaging and the role of laparoscopy for resection in unusual anatomical sites.

## Introduction

Mesenteric cysts are uncommon benign intra-abdominal lesions that arise within the mesentery of the gastrointestinal tract and, in some cases, may extend into the retroperitoneum [1-6]. The condition was first described by Benevieni in 1507 during an autopsy and later documented in detail by Tillaux in 1880. Despite centuries of recognition, mesenteric cysts remain poorly understood and represent a diagnostic and therapeutic challenge.

The incidence is extremely low, estimated to be between one per 100,000 to 250,000 hospital admissions in adults and approximately one per 20,000 in children [1,2,4,5,7-9]. They exhibit a slight female predominance (2:1 ratio) and can occur at any age, although children may present with more acute manifestations. Because of their rarity, most clinicians will encounter very few, if any, cases in their careers.

The pathophysiology remains uncertain. Several theories have been proposed, including congenital malformations of the lymphatic system, failure of embryonic lymphatic spaces to connect with the venous system, obstruction of lymphatic drainage, trauma, infection, and neoplastic proliferation [1,3,5,6]. This uncertainty complicates classification and management, but histopathology provides the most definitive insights.

To clarify the heterogeneity of these lesions, De Perrot et al. proposed the most widely accepted classification system, dividing mesenteric cysts into six histopathological subtypes: lymphatic, mesothelial, enteric, urogenital, mature cystic teratomas, and pseudocysts [9]. This classification not only reflects their diverse origins but also guides clinical expectations and treatment strategies.

The clinical spectrum of mesenteric cysts is broad. Up to half of patients are asymptomatic, with cysts discovered incidentally during imaging or surgery [1,3-7,10]. When symptomatic, the most common complaints are nonspecific abdominal pain, distension, or the presence of a palpable mass. Larger cysts can produce obstructive symptoms, while acute complications such as rupture, torsion, infection, hemorrhage, or bowel obstruction have also been reported. Although uncommon, malignant transformation has been documented in approximately 3% of cases, underscoring the importance of definitive surgical management [1,3,5,6].

Treatment is surgical, as aspiration or marsupialization is associated with high recurrence rates. Complete excision, either open or laparoscopic, is considered curative. The laparoscopic approach, first reported in the early 1990s, has become increasingly favored for its reduced morbidity and faster recovery, provided the cyst’s size and anatomical location are amenable to minimally invasive surgery [3-7,10].

Because of its rarity, it is pertinent to report encountered cases and share the approach used to achieve resolution. In this report, we present the case of a 63-year-old male with a mesothelial mesenteric cyst located near the celiac trunk, an anatomical site that, to our knowledge, has not been previously described in the literature. The patient was successfully treated with laparoscopic excision. By reviewing this case in the context of available literature, we aim to contribute to the understanding of these rare lesions and highlight the feasibility of a minimally invasive approach in unusual anatomical locations.

## Case presentation

A 63-year-old man was referred to our clinic for evaluation of chronic intermittent abdominal pain localized to the left quadrant. The discomfort had been present for several months, was nonspecific in nature, and did not show a clear relation to meals or bowel movements. He denied systemic or acute abdominal symptoms such as distension, nausea, vomiting, changes in bowel habits, or signs suggestive of obstruction or peritonitis.

His past medical history was relevant for type 2 diabetes mellitus, controlled with oral medication (metformin and sitagliptin). He had no prior surgical interventions and reported no family history of gastrointestinal or abdominal diseases.

On clinical examination, his abdomen was soft and non-tender. No palpable mass, organomegaly, or signs of peritoneal irritation were detected. Routine laboratory studies, including complete blood count, renal and hepatic panels, and inflammatory markers, were all within normal limits.

**Figure 1:**
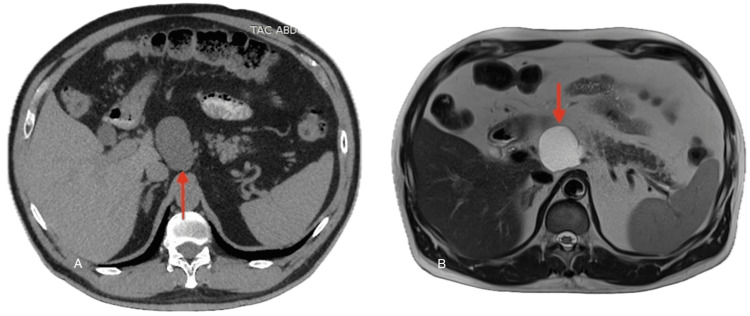
Cross-sectional images of the mesenteric cyst A: CT scan showing mesenteric cyst (red arrow); B: MRI showing mesenteric cyst (red arrow).

Due to the persistent nature of his pain, cross-sectional imaging was performed. An abdominal CT scan (Figure 1A) revealed a well-defined ovoid lesion measuring 56 mm, located close to the origin of the celiac trunk. Subsequent MRI (Figure 1B) demonstrated features consistent with a mesenteric cyst. Importantly, there was no evidence of infiltration or compression of adjacent organs such as the pancreas, liver, or stomach.

**Figure 2:**
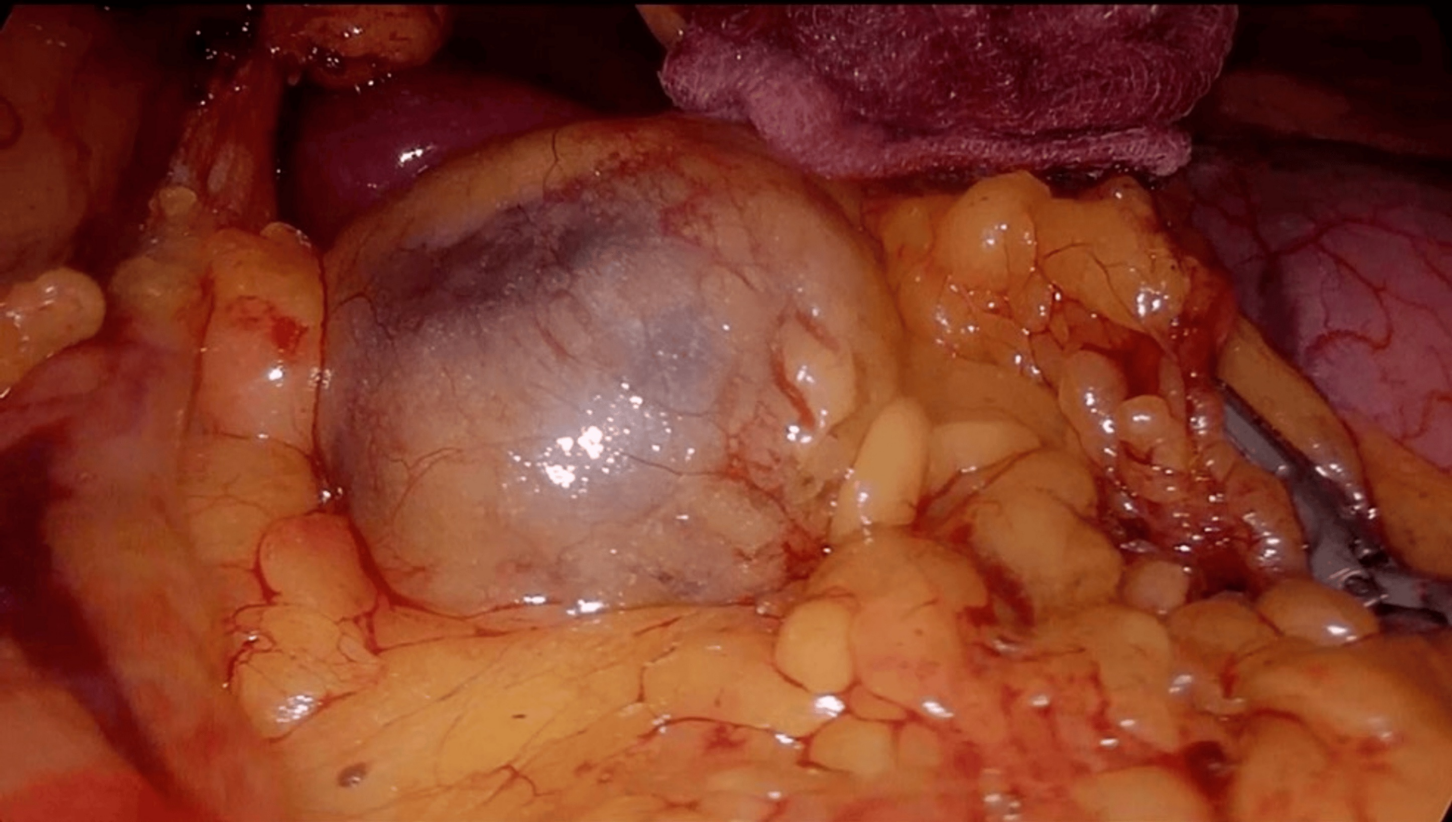
Intraoperative view of the mesenteric cyst

Given these findings, laparoscopic surgical excision was indicated. The patient was placed in the supine position with the surgeon between his legs and the assistant on the right side. Two 10 mm trocars (one umbilical for the camera and one on the left flank) and three 5 mm trocars (two on the right flank and one below the xiphoid bone) were used. Intraoperatively (Figure 2), a cystic mass, measuring approximately 5 cm with apparent fluid content, was identified posterior to the lesser omentum. After careful dissection, the lesser omentum was opened, and the cyst was completely excised without intraoperative complications or need for conversion to laparotomy.

The patient had an uncomplicated postoperative course. The patient tolerated oral intake, mobilized early, and was discharged home on the first postoperative day in good condition.

**Figure 3:**
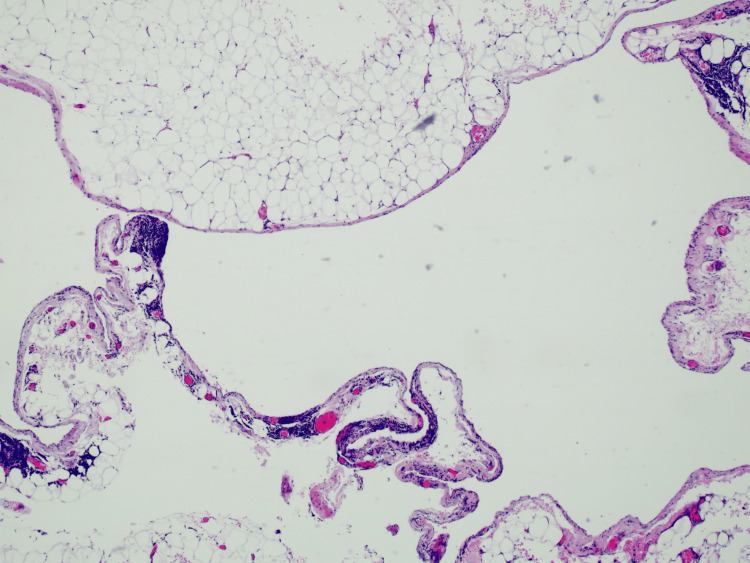
Histopathological analysis consistent with mesenteric cyst

Histopathological analysis confirmed the diagnosis of a benign mesothelial cyst, and cytological examination of the cystic fluid was negative for malignant cells (Figure 3).

At one-month follow-up, the patient remained asymptomatic with no recurrence of abdominal pain or other complaints.

## Discussion

Mesenteric cysts are rare benign intra-abdominal lesions that arise from an anomaly in the embryologic development of the lymphatic system, where incomplete fusion or abnormal communication of lymphatic vessels during mesenteric formation results in a rare cystic lesion. They are most commonly found on the small-bowel mesentery but also reported in the mesocolon or retroperitoneum [1-6,11]. Retroperitoneal extension accounts for approximately 14.5% of cases [11]. The rarity of mesenteric cysts, coupled with their nonspecific presentation, makes diagnosis challenging. Our case is notable for its unique location adjacent to the celiac trunk, which has not been previously documented in the literature.

The clinical manifestations of mesenteric cysts are variable. In approximately 40-50% of cases, they are discovered incidentally [1,4,5]. When symptomatic, the most common complaint is vague abdominal pain, but patients may also present with abdominal distension, constipation, nausea, vomiting, or a palpable abdominal mass [1,6,7]. Complications include rupture, torsion, infection, hemorrhage, or intestinal obstruction, which may result in an acute abdomen and require urgent surgery. In our patient, the only symptom was localized abdominal pain, which illustrates the non-specificity with which these lesions can present.

Imaging plays a central role in the identification and characterization of mesenteric cysts. Ultrasonography is often the first-line modality due to its availability and ability to differentiate cystic from solid lesions. However, CT and MRI provide superior detail, enabling accurate localization, evaluation of size, wall thickness, internal septations, and relation to adjacent structures [9,11,12]. In our case, CT revealed a cystic lesion near the celiac trunk, and MRI further delineated its anatomy, confirming the absence of invasion into adjacent vital structures or other signs of malignancy. Such detailed preoperative imaging was crucial in planning a safe laparoscopic excision.

Definitive treatment of mesenteric cysts is complete surgical excision, as partial excision or drainage is associated with high recurrence rates and increased risk of infection. Historically, laparotomy was the standard approach. However, with advancements in minimally invasive techniques, laparoscopy has become increasingly common since Mackenzie et al. first reported a successful laparoscopic excision in 1993 [10]. Current evidence suggests that laparoscopy is safe and effective, particularly for cysts of moderate size, and offers the advantages of reduced pain, shorter hospitalization, faster recovery, and improved cosmesis [3,12]. In our patient, laparoscopic excision was completed successfully without intraoperative or postoperative complications, supporting its feasibility even in unusual locations near major vascular structures.

Histological evaluation remains the cornerstone for definitive diagnosis. In our case, the lesion was confirmed as a mesothelial cyst, one of the six categories described by De Perrot et al. [9]. Mesothelial cysts are relatively uncommon compared to lymphatic cysts. While most are benign, histological confirmation is important to exclude rare malignant transformation.

Most published cases describe cysts arising from the small-bowel mesentery, commonly in the ileum, and ranging in size from a few centimeters to over 10 cm. Retroperitoneal cases, although rare, have also been described [11]. The majority of cases are managed surgically, with both open and laparoscopic approaches showing excellent outcomes [1-12]. To our knowledge, no cases involving a cyst located adjacent to the celiac trunk have been previously reported, highlighting the uniqueness of this case.

## Conclusions

Although rare, mesenteric cysts should be considered in the differential diagnosis for patients presenting with abdominal pain or a mass. Diagnosis is challenging and typically requires cross-sectional imaging with CT or MRI, as demonstrated in this case. Definitive treatment is complete excision, which can be done safely via laparoscopy, as shown by this case report. The proximity to vital structures can imply more severe symptoms or complicate surgical management. The case presented showed a mesenteric cyst near the celiac trunk, which, to the best of our knowledge, has not yet been reported in the literature.

## References

[1] Singh S, Shukla RK, Gharde P (2023). Mesenteric cyst: a rare entity. Cureus.

[2] Cudia B, D'Orazio B, Calì D, Di Vita G, Geraci G (2020). Lymphatic mesenteric cyst, a rare cause of surgical abdominal pain: case report and review of the literature. Cureus.

[3] Xiao Y, Chaudhari S, Khattak T, Tiesenga F (2022). A rare case of abdominal tumor: mesenteric cyst. Cureus.

[4] Leung BC, Sankey R, Fronza M, Maatouk M (2017). Conservative approach to the acute management of a large mesenteric cyst. World J Clin Cases.

[5] Bhattacharjee A, Kulkarni V, Lamture Y, Nagtode T, Ramteke H (2022). A rare case of a mesenteric cyst. Cureus.

[6] Alqurashi HE, Alaryni AA, Alsairafi RA, Alharbi AM, Alaqla AA (2023). Mesenteric cyst: a case report. Cureus.

[7] Lee DL, Madhuvrata P, Reed MW, Balasubramanian SP (2016). Chylous mesenteric cyst: a diagnostic dilemma. Asian J Surg.

[8] Kurtz RJ, Heimann TM, Holt J, Beck AR (1986). Mesenteric and retroperitoneal cysts. Ann Surg.

[9] de Perrot M, Bründler M, Tötsch M, Mentha G, Morel P (2000). Mesenteric cysts. Toward less confusion?. Dig Surg.

[10] Mackenzie DJ, Shapiro SJ, Gordon LA, Ress R (1993). Laparoscopic excision of a mesenteric cyst. J Laparoendosc Surg.

[11] Saviano MS, Fundarò S, Gelmini R, Begossi G, Perrone S, Farinetti A, Criscuolo M (1999). Mesenteric cystic neoformations: report of two cases. Surg Today.

[12] Mason JE, Soper NJ, Brunt LM (2001). Laparoscopic excision of mesenteric cysts: a report of two cases. Surg Laparosc Endosc Percutan Tech.

